# ^1^H, ^15^N, and ^13^C Resonance Assignment of Human Heat Shock Protein 10

**DOI:** 10.21203/rs.3.rs-6908495/v1

**Published:** 2025-06-25

**Authors:** Abigail Page, Wyatt Hendricks, Marielle A. Wälti

**Affiliations:** University of Arizona

**Keywords:** Hsp10, co-chaperone, Solution NMR, resonance assignment

## Abstract

The human chaperonin system, Hsp60/Hsp10, is essential for maintaining protein homeostasis and is found mainly in mitochondria. Hsp60 forms a bowl-shaped structure that provides an enclosed environment for protein folding, while its co-chaperone, Hsp10, acts as a cap to seal the barrel. This coordinated process is crucial for the proper folding of many unfolded or misfolded proteins, making the Hsp60/Hsp10 complex an indispensable chaperone system. Changes in their expression levels have been linked to diseases such as neurodegenerative disorders and cancer. Although Hsp60 has gained increasing attention, its co-chaperone Hsp10 remains relatively underexplored and has often been assumed to play a passive role. However, emerging studies challenge this view, suggesting that Hsp10 alone may exert regulatory functions within the chaperonin cycle. Here, we present the near-complete NMR backbone assignment of the 102-residue human Hsp10, laying the groundwork for future investigations into its structure, interactions, and roles in facilitating protein folding and preventing aggregation.

## Biological context

The human mitochondrial chaperone system, composed of Hsp60 and its co-chaperone Hsp10, has long been recognized as essential for maintaining proteostasis. Historically, much of our understanding of the human Hsp60/Hsp10 machinery has been adapted from studies of its bacterial homologues, GroEL and GroES, respectively (Bukau & Horwich, 1998; Fenton & Horwich, 1997; Hayer-Hartl et al., 2016; Saibil, 2013). This reliance on the bacterial model has largely been driven by the superior biochemical stability of GroEL, which facilitated its detailed characterization through a variety of structural and functional approaches (Horwich & Fenton, 2020; Roh et al., 2017). However, recent advances have shifted the focus toward the human system itself, leading to the identification of key mechanistic and structural distinctions between human Hsp60 and GroEL (Gomez-Llorente et al., 2020; Levy-Rimler et al., 2002; Nisemblat et al., 2015; Ricci et al., 2016; Vilasi et al., 2014; Wälti et al., 2021). While GroEL is a stable tetradecamer, Hsp60 can exist as a heptamer, tetradecamer, or even as a monomer. The co-chaperone, Hsp10 (or GroES) is typically found as a heptamer composed of seven identical, symmetrical subunits. GroES has previously been assigned both in free solution as well as bound to GroEL (Fiaux, 2002; Mahesh S. Chandak, 2013). Hsp10 has a total molecular weight of approximately 76.5 kDa, with each subunit weighing about 11 kDa. The heptamer of Hsp60 forms a bowl-like structure, which aligns back-to-back in the non-inverted tetradecamer configuration and substrate proteins are captured at the apical domain of Hsp60. Upon ATP binding, Hsp60 undergoes a large conformational change, during which the apical domain moves about 20 degrees upwards and twists about 100 degrees clockwise doubling the volume of the cavity (Clare et al., 2012; Saibil et al., 2013). This structural rearrangement exposes the substrate-binding site, enabling the co-chaperone to bind at the same location. Due to their ability to form a central cavity where protein folding takes place, Hsp60 and Hsp10 are classified as chaperonins. Despite growing interest in the human chaperonin, the co-chaperone Hsp10 has long been considered to play a passive, auxiliary role in the folding cycle—merely capping the Hsp60 complex to facilitate productive encapsulation of substrate proteins.

Genes encoding Hsp60 and Hsp10 are essential, and only a few mutations have been identified. For example, in Hsp10, a very rare mutation—where leucine at position 73 is replaced by phenylalanine—has been associated with infantile spasms (Bie et al., 2016). Emerging evidence challenges this simplistic view, suggesting that human Hsp10 may play a more nuanced role and potentially regulatory function in the chaperonin cycle (David et al., 2013; Larsson et al., 2022; Yeung et al., 2023). Hsp10 has also been found in the cytosol and in secretory granules (Sadacharan et al., 2001; Velez-Granell et al., 1994). Furthermore, according to the Human Protein Atlas (http://www.proteinatlas.org/), Hsp10 is more abundant than Hsp60 (Larsson et al., 2022), and even more abundant than Hsp60 in cancerous environments, supporting the idea that Hsp10 may also have independent functional roles that contribute to both normal cellular health and disease processes.

NMR spectroscopy provides a powerful tool to investigate Hsp10’s role in maintaining protein homeostasis. This assignment will help towards further characterization of Hsp10’s function as a chaperone. Here, we report the nearly complete backbone resonance assignment of the 102-amino acid human Hsp10 co-chaperone. These data lay the groundwork for future investigations into the conformational properties and interaction surfaces of Hsp10 with its substrates and contribute valuable information to the limited structural database on this biologically critical yet underexplored component of the mitochondrial chaperonin system. Our findings aim to catalyze further research into the molecular mechanisms by which Hsp10 contributes to protein homeostasis and chaperonin function.

## Methods and experiments

### Expression of Hsp10

The human cpn10 gene was ordered with codon optimization through GenScript containing a 6xHis tag and a thrombin protease cleavage site. This plasmid was cloned into the pET21d(+) plasmid and transformed into *E. Coli* BL21(DE3) cells and grown on Luria Broth (LB)-agar plates, supplemented with 0.1 mg/mL ampicillin.

#### ^1^ H,^15^N labelled Hsp10

One colony was picked from fresh amplification and grown in 15 mL of ampicillin supplemented LB and grown at 37 °C at 200 rpm for 15–18 hours. The next morning, the 15 mL of cell culture was centrifuged at 5000 rpm for 30 minutes at 25 °C and the pellet was resuspended with minimal media (M9) containing 1 g ^15^N-ammonium chloride as the only nitrogen source. The cells were then transferred to a full liter of ^15^N minimal media supplemented with ampicillin and grown to an OD_600_ of 0.8–1.2, at which point it was induced with 0.5 mM isopropyl-B-D-thiogalactoside (IPTG) and left to continue growing at 37 °C for six hours. The cells were then centrifuged at 4500 rpm for 30 minutes and the pellet was immediately processed according to the protocol described below.

#### ^2^ H,^15^N,^13^C labelled Hsp10

The protocol for expressing deuterated protein was adapted from Dr. Byrd’s lab (Li & Byrd, 2022). Briefly, fifteen colonies were picked from a fresh transformation and grown in 15 mL of LB medium supplemented with ampicillin at 37°C until reaching an OD_600_ of 0.4. The culture was then diluted 1:1 with deuterated minimal medium (^2^H-M9) containing 1 g ^15^N-ammonium chloride and 2 g ^13^C_6_^2^H_7_-glucose. The ^2^H-M9 medium, supplemented with ampicillin, was allowed to grow until an OD_600_ of 0.4 was reached, and then diluted again 1:1. This dilution step was repeated two more times until a final volume of 120 mL was reached. The culture was then incubated at 200 rpm for 15–18 hours. The next morning, the cell culture was centrifuged at 5000 rpm for 30 minutes at 25 °C, and the resulting pellet was resuspended in 1 liter of ^2^H-M9 minimal medium supplemented with ampicillin and ISOGRO-D powder (Sigma-Aldrich). The culture was grown to an OD_600_ of 0.8–1.2, induced with 0.5 mM IPTG, and incubated at 37 °C for six hours. Cells were then harvested by centrifugation at 4500 rpm for 30 minutes.

### Purification of Hsp10

The pellet was resuspended in 40 mL of lysis buffer containing 10 mM imidazole, 300 mM NaCl, 50 mM Tris-HCl pH 8.0. One tablet of cOmplete EDTA free Protease Inhibitor Cocktail tablet (Roche Applied Sciences) and 0.4 g of streptomycin sulfate was added and stirred for 30 minutes at 4 °C. The cells were then homogenized with a cell homogenizer (AVESTIN) at 5 °C. The homogenized sample was centrifuged at 20000 rpm for 25 minutes at 4 °C and the supernatant was collected. The sample was incubated again with 0.4 g of streptomycin sulfate at room temperature for 30 minutes, centrifuged at 4500 rpm for 30 minutes, and filtered through a 0.45 μm cut off filter. The sample was loaded at 1 mL/min onto a 5 mL nickel column (HisTrap^™^ HP, GE Healthcare) that had been equilibrated with buffer A (containing 300 mM NaCl, and 1 M Tris-HCl, pH 8.0) including 10 mM imidazole, using Fast Protein Liquid Chromatography (Bio-Rad). The column was washed by increasing the imidazole concentration to 60 mM in buffer A to remove impurities bound through nonspecific interactions. The protein was then eluted using the same buffer with the imidazole concentration increased to 250 mM. Finally, the column was washed with 1 M imidazole in the same buffer. The sample containing the protein was dialyzed in 200 mM NaCl, 10 mM imidazole, 50 mM Tris HCl pH 8.0, and 1 mM EDTA for four hours at room temperature in a 3.5 MWCO dialysis tubing (Thermo Scientific^™^ SnakeSkin^™^ Dialysis Tubing). The sample was then transferred to a new dialysis bath and thrombin protease (Fisher Scientific) was added to the sample at a ratio of 1:100 w/w and incubated overnight at 4 °C. The next morning, the sample was removed from the dialysis tubing, and 10 mM MgCl_2_ was added to neutralize the chelating effect of EDTA. The sample was loaded onto another 5 mL nickel column previously equilibrated in buffer A. The flow-through, containing the cleaved protein, was collected, and the His-tag was washed from the column using the 1 M imidazole buffer A. The sample was then concentrated using a 30 kDa MWCO Vivaspin concentrator (Sartorius) and injected onto a Superdex 75 (26/60) (Cytiva) size-exclusion column equilibrated in 40 mM sodium phosphate, pH 7.0, to separate Hsp10 from thrombin. SDS-PAGE (4–12% Bis-Tris gel, Invitrogen) and mass spectrometry were used to confirm sample purity. The sample stored in aliquots of 100 μl at 1 mM and − 20 °C.

## NMR spectroscopy

### Solution-state NMR spectroscopy

The ^15^N,^13^C,^2^H-labelled Hsp10 sample was diluted to 300 μM with 40 mM sodium phosphate, pH 7.0. 5% (v/v) D_2_O and 0.03% (v/v) sodium azide (NaN_3_) were added; NaN_3_ served to prevent microbial growth. The sample with a total volume of 350 μL was pipetted into an NMR Shigemi tube. All NMR experiments for assignments were recorded on a Bruker 600 Avance NEO MHz spectrometer equipped with triple resonance TCI cryoprobes optimized for ^1^H detection using TopSpin version 4.0. Most experiments were carried out at 298 K; however, to resolve some overlapping peaks and assure right assignment, measurements were also performed at 303 K. Backbone resonance assignments were achieved using the standard double and triple resonance experiments (Bax & Grzesiek, 1993; Sattler et al., 1999), including 2D ^1^H^15^N HSQC-TROSY (Pervushin et al., 1997), 3D TROSY-HNCA, -HNcoCA, and -HNCACB with 25% non-uniform sampling at 298 K and 80% at 303 K. A 3D [^1^H,^1^H,^15^N] TROSY-NOESY was recorded at 298 K to validate peaks not confirmed with the previous methods (Zhu et al., 1999). An HNCO spectrum was recorded with 80% NUS at 298 K. However, only a few residues produced signals, and thus the spectrum was not useful for assignment purposes other than providing CO chemical shifts for certain residues. Data reconstruction was achieved with the program SMILE (Ying et al., 2017). Spectral data were processed using NMRPipe (Delaglio et al., 1995) and analyzed with CCPNmr Analysis version 3.1.1 (Skinner et al., 2016). Secondary structural elements were predicted with TALOS+ (Shen et al., 2009).

### Assignment and data deposition

**Figure 1:**
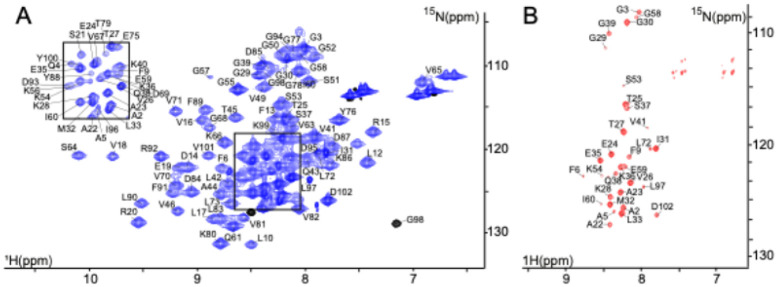
2D spectra of human Hsp10 acquired at 600 MHz and 298 K. (A) 2D ^1^H,^15^N TROSY-HSQC spectrum of 300 mM ^15^N,^13^C,^2^H-labelled human Hsp10 and (B) 2D ^1^H,^15^N TROSY-HSQC of 1 mM ^15^N,^13^C,^1^H-labelled Hsp10 showing the flexible loops of Hsp10. Amide backbone resonances are annotated with the corresponding amino acid one-letter code and residue numbers according to the primary sequence. The black box in A is shown at a lower contour level to reveal the individual peaks. The protein was in 40 mM sodium phosphate at pH 7.0, 5% (v/v) D_2_O, and 0.03% (v/v) NaN_3_.

**Figure 2:**
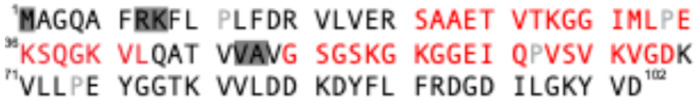
Sequence of the Hsp10 monomer. Residues highlighted in gray depict the unassigned residues, proline residues are shown in gray, and in red are the flexible loops of Hsp10.

Backbone resonance assignments were obtained for the symmetric heptameric Hsp10 complex, at 76.5 kDa, composed of seven identical subunits, each consisting of 102-residues (Fig. 1). The assignments are referenced according to the residue numbering of the Hsp10 monomer and cover 95% of the backbone amide resonances of non-proline residues (Fig. 2). The assignment has been deposited in BMRB under the accession number 53226.

**Figure 3:**
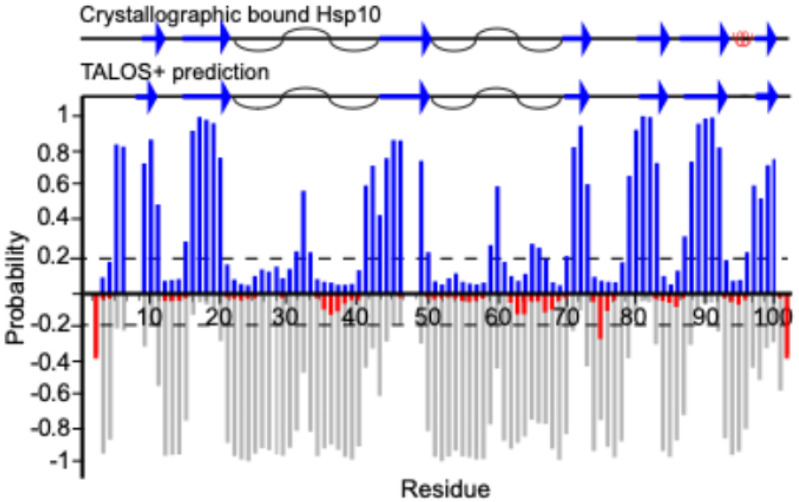
TALOS+ was used to predict the secondary structure of Hsp10. Probabilities for β-sheets are shown in blue, α-helices in red, and disordered regions in grey. The secondary structure elements of Hsp10 are taken from a Hsp60 bound structure from the PDB (code: 6mrc) and is illustrated at the top, where blue arrows represent β-strands, red coils indicate α-helices, and black curves represent the flexible loops. Secondary structure prediction was considered relevant if there were 3 or more residues with structure prediction over 0.2 probability in a row.

Backbone chemical shift values, including ^1^H_N_,^15^N, ^13^Cα, and ^13^Cβ resonances, were utilized as input for secondary structure prediction using TALOS + algorithm (Fig. 3) (Shen et al., 2009). The prediction correlates well with the secondary structural elements found in a structure where Hsp10 is bound to its chaperone Hsp60, taken from 6mrc.pdb (Gomez-Llorente et al., 2020).

Here, we assigned 95% of the amine-containing amino acids of the 76.5 kDa symmetric heptameric Hsp10 co-chaperone. This assignment provides a foundation for future structure–function and dynamics studies of Hsp10 and lays the groundwork for a thorough exploration of its biological relevance.

## Data Availability

The chemical shift data from this study have been deposited in the Biological Magnetic Resonance Data Bank (BMRB) under the accession number 53226.
